# Small intestinal submucosa-derived extracellular matrix bioscaffold significantly enhances angiogenic factor secretion from human mesenchymal stromal cells

**DOI:** 10.1186/s13287-015-0165-3

**Published:** 2015-09-07

**Authors:** Xin Lin, Mikella Robinson, Tye Petrie, Veronica Spandler, W. Douglas Boyd, Claus Svane Sondergaard

**Affiliations:** Department of Surgery, UC Davis Medical Center, Sacramento, CA USA; Department of Surgery, Division of Cardiothoracic Surgery, UC Davis Medical Center, Sacramento, CA USA

## Abstract

**Introduction:**

The in vivo therapeutic effect of mesenchymal stromal cells (MSCs) is currently believed to be tightly linked to their paracrine secretion ability. However, insufficient or imprecise cell delivery, low cell survival and retention post-transplant, along with harsh donor site microenvironments, are major barriers to the clinical success of MSC therapies. Here we tested a small intestinal submucosa (SIS)-derived extracellular matrix (ECM) bioscaffold augmented with MSCs, with the hypothesis that they will facilitate the precise delivery of increased numbers of MSCs therefore improving cell viability and retention.

**Methods:**

In this study, we evaluated the secretion of angiogenic factors from three human MSC lines cultured on SIS ECM. We used human antibody array and enzyme-linked immunosorbent assay to measure the level of angiogenic factors released from MSCs when cultured on SIS ECM or regular tissue culture plastic. We tested MSCs cultured for three different time points.

**Results:**

We found that the SIS ECM culture environment can significantly enhance the release of several angiogenic factors when compared to MSCs cultured on standard tissue culture plastic. Specifically, vascular endothelial growth factor and interleukin-8 secretion was significantly increased at 24, 48 and 72 hours postseeding onto SIS ECM whereas vascular endothelial growth factor release for cells cultured on plastic surface remained the same during these time points. We also observed significant donor to donor variation in cytokine production.

**Conclusions:**

This study demonstrates that MSCs transplanted onto a SIS ECM may greatly increase their therapeutic potential through an increase in pro-angiogenic cytokine release.

**Electronic supplementary material:**

The online version of this article (doi:10.1186/s13287-015-0165-3) contains supplementary material, which is available to authorized users.

## Introduction

Mesenchymal stromal cells (MSCs) are one of the few stem cell types to have reached late stage clinical trials for a variety of indications, including multiple trials as therapeutic agents for ischemic tissue repair [1–5]. In addition to their multipotent differentiation potential, a strong paracrine effect has been proposed as the principal mechanism that contributes to tissue repair [6–9]. The MSC treatment for ischemic heart disease (IHD) has proven especially promising, as the pro-angiogenic, pro-survival, and pro-immunomodulatory paracrine signals released by MSCs can rescue the native cells of an injured heart [10–12].

The stimulation of angiogenesis is of particular importance when treating cardiovascular disease, as it has been shown that improvement in angiogenic support accounts for amelioration of coronary artery disease following bone marrow (BM) cell transplantation [13, 14]. BM-derived MSCs secrete angiogenic factors such as vascular endothelial growth factor (VEGF), hepatocyte growth factor (HGF) and monocyte chemoattractant protein 1 (MCP-1) that are critical for vascular network remodeling [15–17]. It is this physiological mechanism that led to the use of MSCs to treat IHD. Furthermore, interleukin (IL)-8, an inflammatory chemokine with potent pro-angiogenic properties, is upregulated in ischemic injuries and has been shown to promote homing of BM-derived cells to sites of injury. A recent study found that IL-8 induced VEGF production [18] and that this VEGF production in human BM-derived MSCs significantly increased the in vitro angiogenic response compared with basal-secreted VEGF [18]. In the present study we therefore looked at VEGF and IL-8 secretion from MSCs as they are two key angiogenic factors.

The majority of clinical trials using MSCs to treat IHD have used a needle or catheter injection of therapeutic cells freely suspended in liquid carrier solutions. However, it has become apparent that these methods result in poor cell retention and survival, thus reducing therapeutic potential [19]. Naturally derived or synthetic materials have been explored to enhance stem cell survival and retention in vivo [20], including alginate-, hyaluronic acid-, collagen- or extracellular matrix (ECM)-based natural materials and synthetic peptide or polymer-based substances. In the present study, we employed porcine small intestinal submucosa (SIS), an ECM-based natural material, in conjunction with our BM-derived MSCs as an implantable device for tissue repair.

Porcine SIS is commercially available and widely used for tissue remodeling in the clinic for various indications, including vascular and cardiac repair [21–23]. The material is derived from SIS through a process where the ECM is decellularized while still retaining the naturally fibrous and porous nature of the matrix as well as several matrix-associated cytokines [24]. The SIS ECM has been found to be superior to synthetic materials for several indications, as it does not encapsulate when surgically implanted and is gradually remodeled leaving behind organized tissue. Currently, SIS ECM has been used in cardiac repair and pericardial closure in the clinic, but mainly for providing structural support [22]. To maximize its regenerative capability, we propose to recellularize the SIS ECM with BM-derived MSCs, thereby combining the mechanical and biochemical properties of the SIS ECM with the therapeutic capabilities of the MSCs.

The goal of this study was to assess the angiogenic secretome changes of the combined SIS ECM and MSC product when compared to SIS ECM or MSCs alone. In this study, we investigated secretome changes of MSCs after culture on SIS ECM for up to 72 hours. We then focused on two key angiogenic factors, VEGF-A and IL-8, and analyzed their release as a function of time as well as donor origin. Finally, we confirmed the functional relevance of these secreted cytokines in promoting cardiac endothelial cell tube formation on Matrigel, a basement membrane purified from Engelbreth-Holm-Swarm tumor.

## Materials and methods

### MSC culture and characterization

All stem cell experiments were performed in accordance with the guideline by the Institutional Stem Cell Research Oversight committee. Primary human cells are commercially available (Lonza, Walkersville, MD, USA) and their procurement is thus not subject to institution review board oversight.

Culture media (D20) consisted of Dulbecco's modified Eagle's medium high glucose, 1 % L-glutamine 200 mM, 1 % penicillin-streptomycin (all Hyclone Laboratories, South Logan, UT, USA) and 20 % fetal bovine serum (Atlanta Biologicals, Lawrenceville, GA, USA). Porcine SIS-derived ECM was from Cook Biotech (West Lafayette, IN, USA).

Human MSCs from three different donors were isolated from BM samples obtained from Lonza and tested for putative MSC markers and tri-lineage differentiation potential [25]. Briefly, cells were expanded in D20 media before plating in adipogenic, osteogenic or chondrogenic differentiation media according to the manufacturer’s specifications (Lonza). Cell differentiation was visualized by staining with Alizarin Red, Oli Red O, and Alcian Blue for osteocytes, adipocytes and chrondrocytes, respectively. Flow cytometric analysis was performed on cell samples stained with CD73 (Biolegend, San Diego, CA, USA), CD90, CD34, CD19, HLA-DR (BD Biosciences, San Jose, CA, USA), CD105 (Abcam, Cambridge, MA, USA), CD45, and CD14 (AbD Serotec, Raleigh, NC, USA). Relevant isotype controls were run in parallel. Samples were run on a FC500 and analyzed using software supplied by the manufacturer (Beckman Coulter, Brea, CA, USA).

### Condition media collection

Cells were cultured in D20 in a 96-well plate, either on a 6-mm diameter circular SIS ECM disc or directly on the surface of tissue culture plate. The seeding density for both conditions was 25,000 cells/well. Conditioned media was collected every 24 hour for 3 days for both conditions. Fresh D20 culture media was added after each time point for the next conditioned media collection. Two control conditioned media groups were collected in parallel: a) “ECM only” control consisting of an unseeded 6-mm diameter SIS ECM disk incubated with D20 culture media for 24 hours followed by fresh media replacement and collection every 24 hours; and b) “media only” control consisting of fresh D20 culture media collected after 24 hours of incubation. Individual samples are identified by donor number, time point and culture condition, e.g., conditioned media collected from donor 1 cells growing on matrix for 24 hours is named “Cells + SIS ECM D1 24 h”.

A subset of cells was transduced with a lentiviral vector harboring the enhanced green fluorescent protein (eGFP) gene in the presence of 2 mg/ml protamine sulfate. Transgene expression was verified on a Motic AE30-31 inverted fluorescence microscope at the magnification indicated using a Moticam Pro252A imaging system (both Motic Instruments, Canada) and further quantified on a FC500 and analyzed using software supplied by the manufacturer (Beckman Coulter, Brea, IN, USA). Transduction efficiencies of >90 % eGFP positivity were routinely obtained (not shown). Cell growth over time was estimated by plating 25,000 eGFP overexpressing MSCs on either SIS ECM or tissue culture plastic as described above and relative eGFP fluorescence was recorded at 24, 48, and 72 hours postseeding on a Spectramax i3 imaging system (Molecular Devices, Sunnyvale, CA, USA). Fluorescent values were corrected for background signal from media and SIS ECM samples alone run in parallel.

### MSC angiogenic factor profiling by human angiogenesis array

Conditioned media collected from donor 1 at the 72 hour time point of all four culture conditions was assayed for the expression profiles of angiogenesis-related proteins. An R&D systems (Minneapolis, MN, USA) antibody array of 55 angiogenesis-related proteins was used in this assay (Table 1). The LI-COR (Lincoln, NE, USA) protocol for human angiogenesis array kit from the vendor was followed for this assay. Array data were analyzed by LI-COR image studio lite ver4.0 software.

**Table 1:** List of targets and controls of the human angiogenesis array

Coordinate	Target
A1, A2	Reference Spots
A5, A6	Activin A
A7, A8	ADAMTS-1
A9, A10	Angiogenin
A11, A12	Angiopoietin-1
A13, A14	Angiopoietin-2
A15, A16	Angiostatin/Plasminogen
A17, A18	Amphiregulin
A19, A20	Artemin
A23, A24	Reference Spots
B1, B2	Coagulation factor III
B3, B4	CXCL16
B5, B6	DPPIV (CD26)
B7, B8	EGF
B9, B10	EG-VEGF
B11, B12	Endoglin (CD105)
B13, B14	Endostatin/collagen XVIII
B15, B16	Endothelin-1
B17, B18	FGF-1
B19, B20	FGF-2
B21, B22	FGF-4
B23, B24	FGF-7
C1, C2	GDNF
C3, C4	GM-CSF
C5, C6	HB-EGF
C7, C8	HGF
C9, C10	IGFBP-1
C11, C12	IGFBP-2
C13, C14	IGFBP-3
C15, C16	IL-1β
C17, C18	IL-8
C19, C20	LAP (TGF-β1)
C21, C22	Leptin
C23, C24	MCP-1
D1, D2	MIP-1α
D3, D4	MMP-8
D5, D6	MMP-9
D7, D8	NRG1-β1
D9, D10	Pentraxin 3 (PTX3)
D11, D12	PD-ECGF
D13, D14	PDGF-AA
D15, D16	PDGF-AB/PDGF-BB
D17, D18	Persephin
D19, D20	Platelet Factor 4 (PF4)
D21, D22	PlGF
D23, D24	Prolactin
E1, E2	Serpin B5
E3, E4	Serpin E1
E5, E6	Serpin F1
E7, E8	TIMP-1
E9, E10	TIMP-4
E11, E12	Thrombospondin-1
E13, E14	Thrombospondin-2
E15, E16	uPA
E17, E18	Vasohibin
E19, E20	VEGF
E21, E22	VEGF-C
F1, F2	Reference Spots
F23, F24	Negative Control

### ELISA analysis of VEGF and IL-8 secretion under different culture conditions from three donors

VEGF-A (denoted VEGF only in the following) and IL-8 were detected by enzyme-linked immunosorbent assay (ELISA) in conditioned media from three different human donors seeded on plastic or SIS ECM, or from control samples collected at 24, 48 or 72 hours, as described above. Human VEGF and IL-8 ELISA kits were from R&D systems and assays were carried out following the manufacturer’s description. Each datum point was analyzed in duplicate per ELISA assay, and all ELISA experiments were repeated three times.

### In vitro angiogenic assay

Human microvascular endothelial cells (cardiac) (HMVEC-C) and microvascular endothelial cell growth medium-2 (EGM-2 MV) culture media plus bullet kit were purchased from Lonza. For the in vitro angiogenesis assay, Matrigel for tube formation was purchased from Corning (Corning, NY, USA). In vitro tube formation assays were carried out following manufacturer’s instruction. Briefly, HMVEC-Cs were added onto Matrigel reduced growth factor basement membrane extract in a 96-well plate. Four different types of conditioned media were collected from donor 1 cells at 24 hours in HMVEC-C basal media (EGM-2 MV media + 5 % fetal bovine serum). These were added into each well to observe tube formation of HMVEC-C in different culture conditions. EGM-2 MV complete culture media (EGM-2 MV media + bullet kit) was used as a positive control. Each datum point was averaged from 12 samples assayed on a Zeiss D1 Observer (Lonza, Walkersville, MD, USA) inverted microscope, and the images were blindly analyzed with the ImageJ Angiogenesis Analyzer (Zeiss Microscopy, Pleasanton, CA, USA; Image J, U. S. National Institutes of Health, Bethesda, Maryland, USA) for length of tubes formed, number of branches formed, number of junctions formed, and number of nodes formed.

### Statistics

All data are expressed as mean ± standard deviation. Statistical analysis was performed using student’s *t*-test with Holm-Bonferroni correction. A *p* < 0.05 was taken to be significant.

## Results

### Human MSCs can be seeded and cultured on SIS ECM at high density

**Fig. 1:**
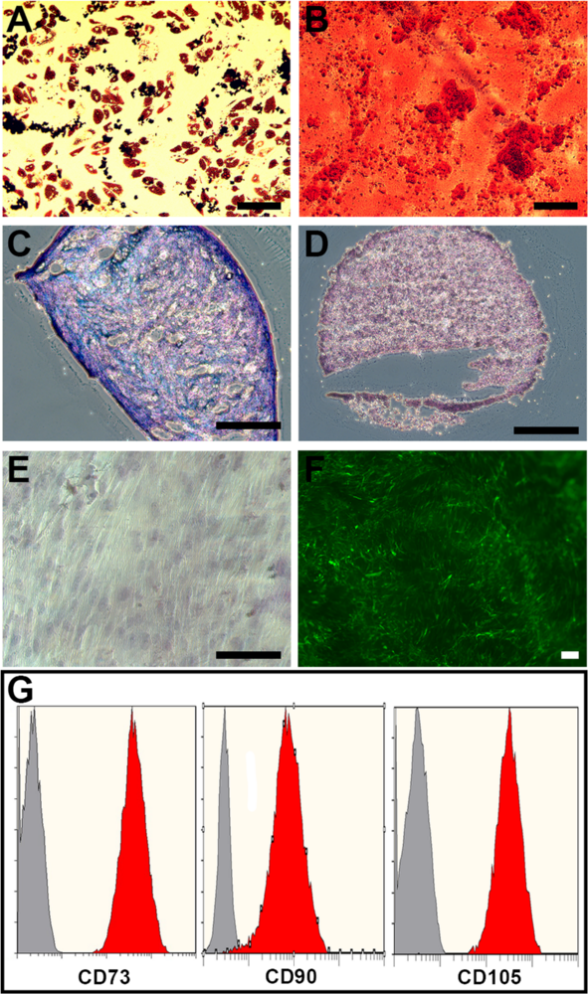
Characterization of human MSCs cultured on SIS ECM. Human MSCs were characterized by tri-lineage induction, cell surface marker expression and eGFP transgene expression. Cells could readily be induced along the adipogenic (**a**), osteogenic (**b**) and chondrogenic lineage (**c**). Non-induced cells remained undifferentiated (**d**, **e**). **f** A subset of MSCs were transduced with eGFP and seeded onto SIS ECM. **g** MSCs stained positive by flow cytometry for the putative MSC surface markers CD73, CD90 and CD105 (*red curve*). Isotype control stained cells were uniformly negative (*grey curve*). Scale bars: **a**, **b** and **f** = 200 μm; **c**–**e** = 100 μm

Human BM-derived MSCs were isolated from three individual donors and screened for tri-lineage differentiation potential (Fig. 1a–e) and the presence of putative MSC cell surface markers (Fig. 1g).

We transduced a subset of MSCs with a lentiviral vector carrying the eGFP gene to allow visualization of the MSCs once seeded onto the SIS ECM. The MSCs readily attached to the SIS ECM (Fig. 1f) and remained viable for up to 7 days in culture as evident from the continued expression of eGFP. Throughout the present study we used a seeding density of 25,000 cells per well or per 6-mm matrix sample.

### Differential expression of angiogenesis factors from MSCs cultured on matrix and on plastic

**Fig. 2:**
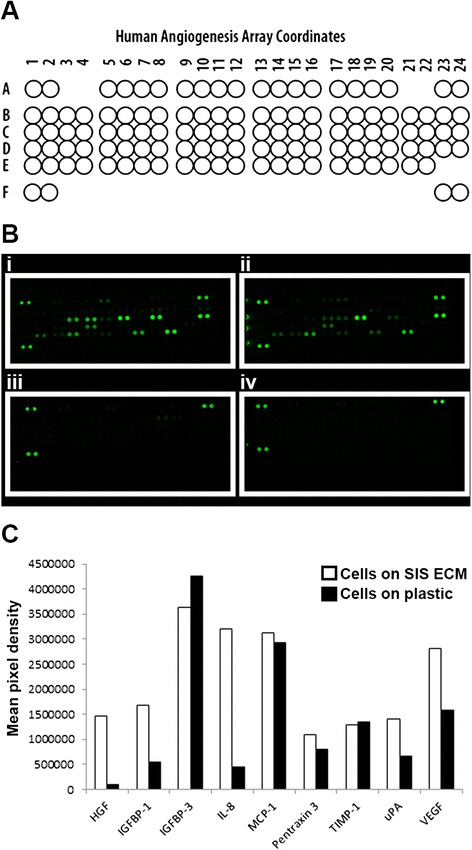
Human angiogenesis array data. **a** Human angiogenesis array coordinates. **b** Scanned images of four human angiogenesis array blots. (i) Conditioned media from human MSCs cultured on matrix for 72 hours. (ii) Conditioned media from human MSCs cultured on tissue culture plate plastic surface for 72 hours. (iii) Matrix control, cell cultured media incubated with only matrix. (iv) Media control, cell culture media only. **c** Comparison of difference in secretion of select analytes from MSCs on matrix or on plastic. Values represent the mean of fluometric intensity measurements of duplicate dots of stained membranes depicted in (**b**). *ECM* extracellular matrix, *HGF* hepatocyte growth factor, *IGFBP* insulin-like growth factor binding protein, *IL* interleukin, *MCP* monocyte chemoattractant protein, *SIS* small intestinal submucosa, *TIMP* tissue inhibitor of metalloproteinase, *uPA* urokinase-type plasminogen activator, *VEGF* vascular endothelial growth factor

Conditioned media was collected from donor 1 cells cultured for 72 hours on either SIS ECM matrix or standard tissue culture plastic. Media incubated for 72 hours with or without matrix served as controls. Cytokine arrays were used to measure the expression profiles of 55 angiogenic-related proteins (Table 1 and Fig. 2). We found only minimal background signal in the medium only control, whereas media incubated with matrix only reacted weakly for fibroblast growth factor (FGF)-1 and FGF-2. In media from cells cultured on matrix, we found relatively high expression levels of the following nine analytes: HGF, insulin-like growth factor binding protein (IGFBP)-1, IGFBP-3, IL-8, MCP-1, Pentraxin 3, tissue inhibitor of metalloproteinase (TIMP)-1, urokinase-type plasminogen activator (uPA) and VEGF. Cells on matrix express higher levels of six of these nine analytes than cells cultured on plastic. Cells cultured on matrix express 14-fold HGF, 3-fold IGFBP-1, 7-fold IL-8, 1.4-fold pentraxin 3, 2-fold uPA and 1.8-fold VEGF when compared to cells cultured on plastic. MCP-1 and TIMP-1 are expressed in similar concentrations by cells regardless of culture conditions. Only IGFBP-3 expression levels are slightly lower in samples from cells cultured on matrix than samples from cells culture on plastic (Fig. 2).

### Quantitative measurement of VEGF and IL-8 by ELISA

**Fig. 3:**
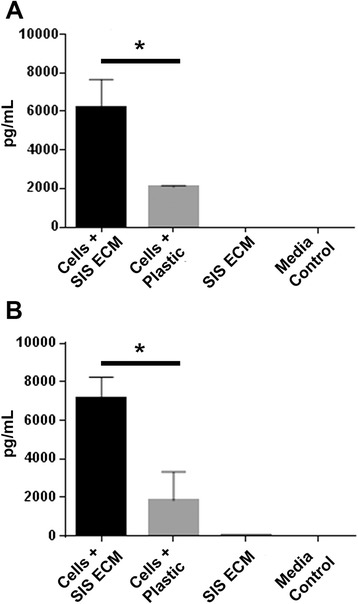
ELISA measurement of VEGF and IL-8 released from donor 1 MSCs after culture for 48 hours. Human MSCs from donor 1 were seeded on small intestinal submucosa (SIS) extracellular matrix (ECM) or cell culture plastic, and culture supernatant was used to quantify **a** VEGF and **b** IL-8 secretion at 48 hours postseeding. Media and SIS ECM only samples were used as controls. Error bars represent SD of three biologic replicates with internal duplicates. *p < 0.05

We further analyzed two key angiogenic factors, VEGF and IL-8, which showed enhanced expression from cells cultured on matrix. We first analyzed conditioned media from donor 1 MSCs cultured for 48 hours either on matrix or on plastic and compared this to 48-hour matrix and media controls by ELISA. We confirmed the increased cytokine production of both VEGF and IL-8 from MSCs cultured on matrix. At 48 hours, the level of VEGF was significantly higher (6210.8 ± 1448.9 pg/ml) from cells cultured on matrix as compared to cells cultured on plastic (2088.4 ± 63.8 pg/ml, *p* = 0.008; Fig. 3a), which corresponds to an approximate threefold increase in VEGF from cells cultured on matrix. Similarly, we found that the IL-8 level at the same time point was 7117.7 ± 1127.4 pg/ml from cells cultured on matrix, which was about fourfold higher than IL-8 from cells cultured on plastic (1844.7 ± 1488.3 pg/ml, *p* = 0.008; Fig. 3b). These data thus closely mirrored what we observed in the angiogenic array blot. Donors 2 and 3 also showed the same expression pattern of VEGF and IL-8 (Additional file 1: Figure S1). In the matrix and media controls, the amount of either of cytokines was below detectable levels (Fig. 3).

### Temporal expression pattern of VEGF and IL-8 in conditioned media

**Fig. 4:**
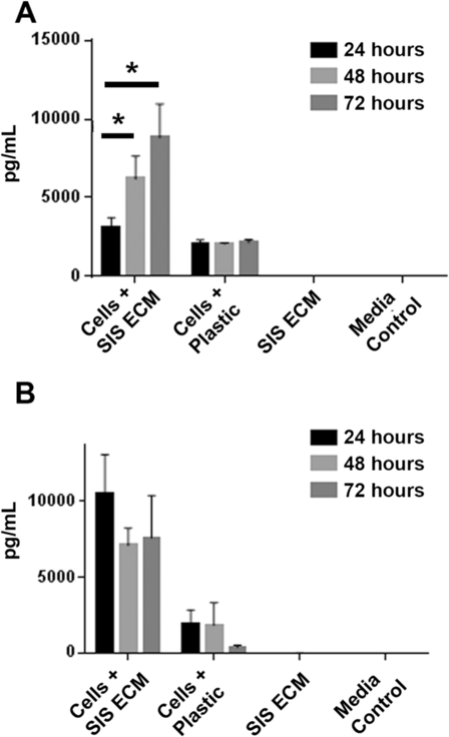
ELISA measurement of VEGF and IL-8 release from donor 1 MSCs at three different time points. Human MSCs from donor 1 were seeded on small intestinal submucosa (SIS) extracellular matrix (ECM) or cell culture plastic and culture supernatant was used to quantify **a** VEGF and **b** IL-8 secretion at 24, 48 and 72 hours postseeding. Media and SIS ECM only samples were used as controls. Error bars represent SD of three biologic replicates with internal duplicates. *p < 0.05

We examined how VEGF and IL-8 levels change over time by comparing donor 1 MSCs cultured for 24, 48 and 72 hours on matrix or on tissue culture plastic. We again included matrix and media controls at all three time points. VEGF levels increased as time progressed for MSCs cultured on matrix, whereas VEGF levels stayed the same or at a lower level when MSCs were cultured on plastic (Fig. 4a). For cell cultures on matrix, we found a significant difference in VEGF levels between the 24-hour and 48-hour time points (*p* = 0.028) and also between the 24-hour and 72-hour time points (*p* = 0.011) (Fig. 4a). IL-8 levels appeared to decrease slightly at 48 hours and 72 hours compared to the 24-hour time point when cultured on matrix, although the difference between these three time points were not significant. MSCs cultured on plastic had much lower IL-8 levels overall which remained the same at 24 and 48 hours with a trend towards a decline at 72 hours (Fig. 4b). For donors 2 and 3, VEGF levels also increased as time progressed for MSCs cultured on matrix. For donor 2, the increase among the three time points are not statistically significant. However, for donor 3, we also found a significant difference in VEGF levels between the 24-hour and 48-hour time points (*p* = 0.043) and also between the 24-hour and 72-hour time points (*p* = 0.009) (Additional file 2: Figure S2A-B). IL-8 levels varied in three time points for MSCs cultured on matrix and the difference is not statistically significant (Additional file 2: Figure S2C-D).

### Cell growth over time

To confirm that the difference in cytokine expression between samples generated from cells seeded on tissue culture plastic and cells seeded on SIS ECM was not due to differences in cell proliferation rates, we analyzed growth rates under the two culture conditions for up to 72 hours postseeding.

**Fig. 5:**
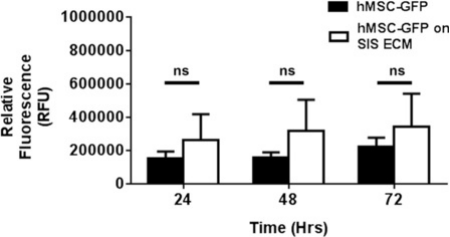
Seeding on small intestinal submucosa (SIS) extracellular matrix (ECM) or culture plastic does not affect cell growth rates. Human mesenchymal stromal cells (hMSCs) from donor 1 overexpressing enhanced green fluorescent protein (GFP) were seeded onto SIS ECM or tissue culture plastic and relative fluorescence intensity was measured after 24, 48 and 72 hours postseeding. Media and SIS ECM only controls were used to correct for background fluorescence. Error bars represent SD of six replicates. *ns* nonsignificant

We seeded eGFP overexpressing MSCs at the same densities as employed above and analyzed green fluorescence as a marker of relative cell density at 24, 48, and 72 hours postseeding. As can be seen in Fig. 5, we found that there was no significant difference in cell density between cells seeded on SIS ECM versus cells seeded on culture plastic at either of the three time points. This confirms that the observed differences in cytokine levels were not due to differential cell growth rates relative to seeding method. Using MSCs from donor 2, we confirmed that there was no difference in green fluorescence between the different time points (Additional file 3: Figure S3), neither did we observe any difference between donor 1 and donor 2 at any of the three time points (data not shown).

### Donor variability of VEGF and IL-8 release

**Fig. 6:**
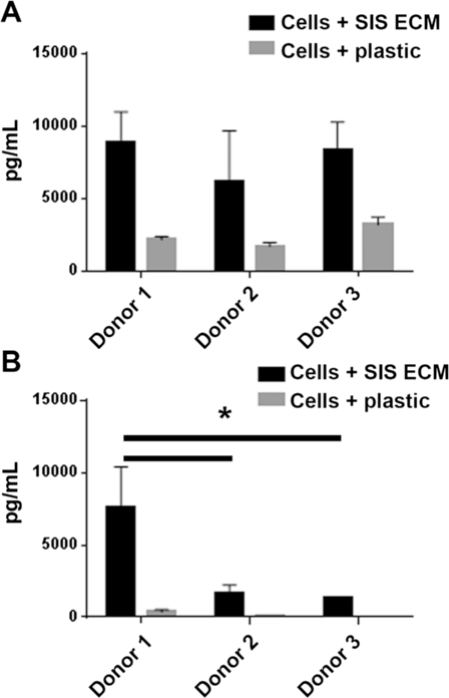
ELISA measurement of VEGF and IL-8 release from MSCs from three human donors after culture for 72 hours. Human MSCs from donors 1, 2 and 3 were seeded on small intestinal submucosa (SIS) extracellular matrix (ECM) or cell culture plastic and culture supernatant was used to quantify **a** VEGF and **b** IL-8 secretion at 72 hours postseeding. Media and SIS ECM only samples were used as controls. Error bars represent SD of three biologic replicates with internal duplicates. *p < 0.05

We compared VEGF and IL-8 release from three human donors at the 72-hour time point in culture supernatant from cells grown on matrix or on tissue culture plastic. We found that donor 1 has the highest secretion of IL-8 and VEGF among three donors when MSCs were cultured on matrix. For IL-8 we found that donor 1 released significantly higher levels as compared to donor 2 and 3 (*p* = 0.018 for donor 1 versus donor 2; *p* = 0.022 for donor 1 versus donor 3). For VEGF, however, the difference among the three donors was not significant (Fig. 6). When MSCs were cultured on plastic, VEGF and IL-8 levels were much lower overall, and donor-to-donor variations were not statistically significant (Fig. 6).

### In vitro angiogenesis tube formation

**Fig. 7:**
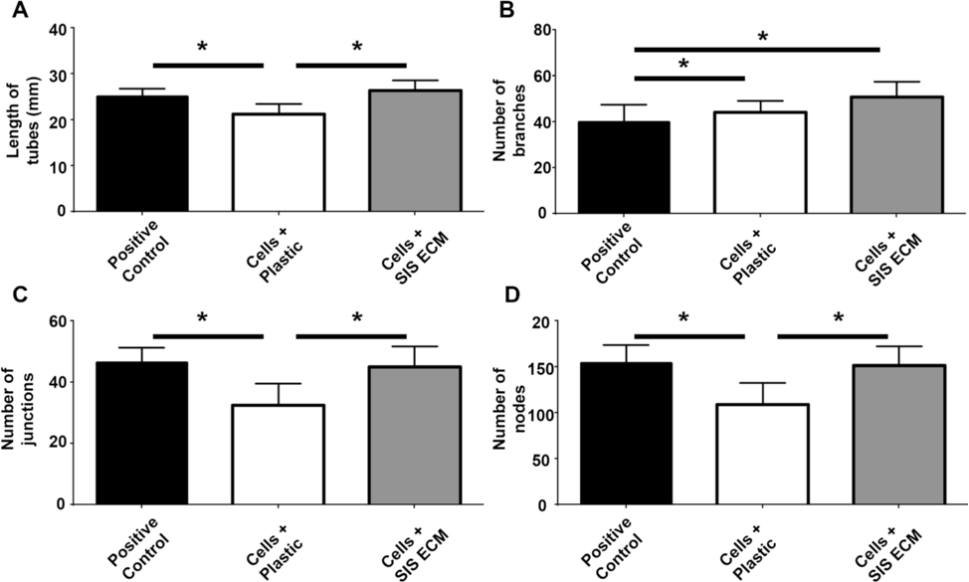
In vitro tube formation using HMVEC-C. HMVEC-C were incubated with conditioned media from MSCs cultured for 24 hours on small intestinal submucosa (SIS) extracellular matrix (ECM) or plastic tissue culture plates as indicated and angiogenic response was evaluated from photomicrographs by calculating **a** total tube length, **b** number of branches, **c** number of junctions, and **d** number of nodes. Control samples received complete HMVEC-C culture media. Each group is represented by the mean of 12 samples and error bars represent SD. *p < 0.05

Angiogenic potential was assessed in vitro with a tube formation assay. HMVEC-C grown on Matrigel were incubated with four different kinds of condition media collected as described above in HMVEC-C basal media, in addition to a positive control of HMVEC-C complete culture media alone. Our data indicate that condition media collected from human MSCs cultured on SIS ECM for 24 hours and human MSCs cultured on plastic for 24 hours can promote endothelial cell tube formation. However, the tube network formation is more extensive in condition media collected from MSCs cultured on matrix, as shown by the significantly increased length of tubes formed (*p* < 0.01; Fig. 7a), number of branches formed (*p* < 0.05; Fig. 7b), number of junctions formed (*p* < 0.01; Fig. 7c), and number of nodes formed (*p* < 0.01; Fig. 7d). Moreover, media from MSCs cultured on SIS ECM performed as well or better than the positive control (not significant for total length, number of junctions and length of nodes; *p* = 0.0176 for number of branches) whereas conditioned media from MSCs cultured on plastic performed worse than the positive control for all outcome measures except number of branches (Fig. 7; *p* < 0.05 for total length of tubes, total length of nodes and total number of junctions and *p* = 0.95 for total number of branches). Two controls, HMVEC-C basal media incubated with only SIS ECM and HMVEC-C basal media did not promote structured tube formation (data not shown). These data demonstrate that the paracrine factors released from our human donor MSCs are functional and that seeding MSCs onto SIS ECM significantly increases their angiogenic potential.

## Discussions

Cell-based regenerative therapy has been intensely studied since the late 1990s, both in pre-clinical and clinical studies. While many therapies have proven to be both safe and efficacious, improvements in key outcome measures in many clinical trials have been modest at best. This paucity in robust regeneration has been attributed in part to poor cell survival and retention at the site of transplantation and a gap in understanding of the therapeutic mechanism of action. In the present report, we explore an alternative approach to delivering therapeutic MSCs by seeding onto an implantable acellular SIS ECM scaffold. We show that human MSCs cultured in vitro on the SIS ECM has an improved angiogenic potential compared to cells cultured under normal plastic adherent conditions, potentially indicating a method for improving survival and retention of the donor cells post-transplant and elucidating a potential method for improving angiogenesis in vivo. Furthermore, we demonstrate that this SIS ECM adherence-dependent difference in secretion of angiogenic factors is both time- and donor-dependent, further highlighting critical parameters of optimization of therapeutic efficacy in vivo.

In this study, we isolated human BM-derived MSCs from three donors. We characterized MSCs by the criteria as recommended by the Mesenchymal and Tissue Stem Cell Committee of the International Society for Cellular Therapy [25] and additionally confirmed a normal karyotype for all three donors (data not shown). Donor to donor variability for MSCs and how it impacts study outcomes is rarely assessed. Here we found significant donor to donor variability in VEGF and IL-8 release. This observation is consistent with findings by others who have reported significant donor to donor differences in differentiation potential and gene expression levels of MSCs [26, 27]. The fact that we also demonstrate significant differences in angiogenic secretome levels further emphasizes the importance of using, for example, cytokine release as screening criteria to select optimal donor cell populations for implantation.

While we found that the pattern of increased secretion of VEGF and IL-8 upon culture on SIS ECM was consistent for the three donors presently analyzed, we cannot formally extend that conclusion to also include the other cytokines evaluated in our array experiment, as these data currently represent only a single donor. Moreover, the present array data are also not meant to be exhaustive with respect to factors other than IL-8 and VEGF that may be involved in improved angiogenic signaling [28]. Indeed we identified several factors that would be interesting candidates for future work; e.g., we found HGF, which is known to work in close concert with VEGF to induce angiogenesis, to also be highly upregulated from MSCs seeded on SIS ECM [29, 30].

Expansion and differentiation potentials of MSCs are known to be dependent on the extracellular environment, i.e., modulation of matrix stiffness can direct human MSCs along muscle or bone lineages [31]. Similarly, various matrices have been explored for engineering of bone or cartilage constructs for implantation [32–34], again focusing on differentiating donor MSCs into functional effector cells. Less is known about the effect of undifferentiated cells when cultured on acellular scaffolds in the context of harnessing the paracrine properties of MSCs for therapeutic application. Here, we seeded human MSCs on SIS ECM to see if the ECM matrix would alter the paracrine secretion ability of MSCs during culture. Using a human angiogenesis array, we demonstrated that culturing human MSCs on SIS ECM enhanced key angiogenic factor release when compared to human MSCs cultured on a regular cell culture plastic surface. We then used ELISA to confirm this finding while focusing on two important angiogenic factors, VEGF and IL-8, which showed prominent differential expression in our array study. This detailed analysis showed that at 48 and 72 hours, VEGF and IL-8 secretion from MSCs cultured on matrix were significantly higher than from cells cultured on plastic. These findings are important for two main reasons when MSCs are used therapeutically for their paracrine effects on angiogenesis. First, MSCs are typically cultured on plastic and delivered in a single-cell suspension, with pre-implant potency testing being determined from in vitro levels of secreted angiogenic cytokines [35–37]. Based on the present results, potency as measured by pre-implantation levels of secreted angiogenic cytokines from cells grown on plastic would, however, grossly under-represent the true in vivo therapeutic potential as the MSCs significantly alter their secretome profile based upon their growth substrate. Second, the fact that we found a significant increase in secretion of key angiogenic factors in response to in vitro culture on SIS ECM highlights an opportunity for increasing the therapeutic potential of MSCs by transplantation of cell-seeded matrices rather than single-cell suspensions.

We also studied if cytokine release changed when MSCs were cultured for 24, 48 or 72 hours. Our data demonstrates an increase in VEGF secretion over time for cells grown on matrix but not on plastic. Of note, we found that the temporal increase in cytokine production was not due to different levels of cell proliferation in that no significant difference was observed in cell density when comparing SIS ECM and cell culture plastic at individual time points. Collectively, these data therefore indicated that the SIS ECM itself provided an optimized culture environment for MSCs resulting in the release of more cytokines that are beneficial for in vivo angiogenesis. Indeed, culturing MSCs on the SIS ECM for up to 72 hours, as documented in the present study, may serve as a beneficial approach to preconditioning the MSCs-augmented ECM patch before implantation.

This study specifically emphasizes porcine SIS ECM bioscaffold and its effect on angiogenic-related cytokine release from human MSCs. However, our observations are likely to extend to ECM derived from other organs and other species that present a three-dimensional structure and biochemical makeup that is beneficial for MSCs and other cell types. We speculate that this may then provide an environmental niche that better approximates the physiological conditions in vivo. These benefits may also not be limited to angiogenic factor release; other factors, such as immunomodulatory and anti-inflammatory cytokines as well as those responsible for anti-apoptotic and antimicrobial properties of MSCs, might also be enhanced.

## Conclusion

This study demonstrates that human MSCs increase their secretion of pro-angiogenic factors in response to seeding onto SIS ECM as compared to culture on normal tissue culture plastic. Specifically, VEGF and IL-8 levels were found to increase over time resulting in as much as a three- to fourfold difference in secreted protein levels at 48 hours postseeding onto SIS ECM compared to control cells. While the pattern of differential cytokine levels was consistent across three different donors, we found significant donor to donor variability, thus highlighting the need for careful screening on potential therapeutic MSC cell populations. Collectively these results demonstrate that transplantation of MSCs on a SIS ECM support may hold great potential for regenerative applications where an increase in therapeutic potential through an increase in pro-angiogenic cytokine release is essential.

## Additional files

Additional file 1: Figure S1. ELISA measurement of VEGF and IL-8 from donors 2 and 3 MSCs after culture for 48 hours. Human MSCs from donors 2 and 3 were seeded on SIS ECM or cell culture plastic and culture supernatant was used to quantify VEGF secretion from donor 2 (a) and donor 3 (b) and IL-8 from donor 2 (c) and donor 3 (d) at 48 hours postseeding. Media and SIS ECM only samples were used as controls. Error bars represent SD of three biologic replicates with internal duplicates. **p* < 0.05. (TIFF 4250 kb) Additional file 2: Figure S2. ELISA measurement of VEGF and IL-8 release from donors 2 and 3 MSCs at three different time points. Human MSCs from donors 2 and 3 were seeded on SIS ECM or cell culture plastics and culture supernatant was used to quantify VEGF secretion from donor 2 (**a**) and donor 3 (**b**) and IL-8 from donor 2 (**c**) and donor 3 (d) at 24, 48 and 72 hours postseeding. Error bars represent SD of three biological replicates with internal duplicates. **p* < 0.05. (TIFF 4707 kb) Additional file 3: Figure S3. Seeding on SIS ECM or culture plastic does not affect cell growth rates. MSCs from donor 2 overexpressing eGFP were seeded onto SIS ECM or tissue culture plastic and relative fluorescence intensity was measure after 24, 48 and 72 hours postseeding. Media and SIS ECM only controls were used to correct for background fluorescence. Error bars represent SD of six replicates. *ns* non-significant, *RFU* relative fluorescent units. (TIFF 1443 kb)

## Abbreviations

BM: Bone marrow
ECM: Extracellular matrix
eGFP: Enhanced green fluorescent protein
EGM-2 MV: Microvascular endothelial cell growth medium-2
ELISA: Enzyme-linked immunosorbent assay
FGF: Fibroblast growth factor
HGF: Hepatocyte growth factor
HMVEC-C: Human microvascular endothelial cells (cardiac)
IGFBP: Insulin-like growth factor binding protein
IHD: Ischemic heart disease
IL: Interleukin
MCP-1: Monocyte chemoattractant protein 1
MSC: Mesenchymal stromal cell
SIS: Small intestinal submucosa
TIMP: Tissue inhibitor of metalloproteinase
uPA: urokinase-type plasminogen activator
VEGF: Vascular endothelial growth factor
