# Supplementary figures and images for: Small intestinal submucosa-derived extracellular matrix bioscaffold significantly enhances angiogenic factor secretion from human mesenchymal stromal cells
Source: Stem Cell Res Ther. 2015 Sep 7;6(1):164. doi: 10.1186/s13287-015-0165-3 (PMC4562125; doi:10.1186/s13287-015-0165-3)

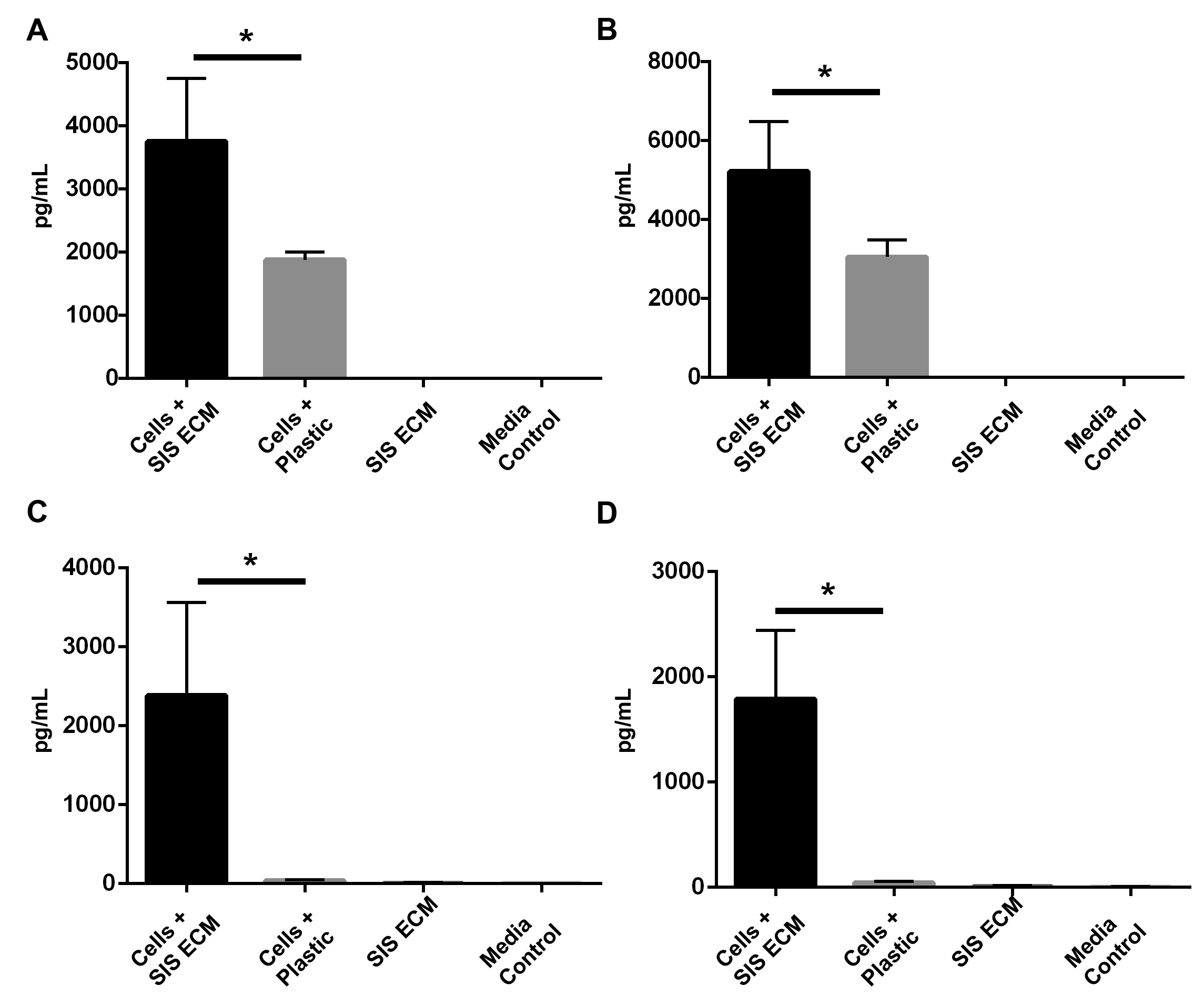

Supplement: Additional file 1: Figure S1. — ELISA measurement of VEGF and IL-8 from donors 2 and 3 MSCs after culture for 48 hours. Human MSCs from donors 2 and 3 were seeded on SIS ECM or cell culture plastic and culture supernatant was used to quantify VEGF secretion from donor 2 (a) and donor 3 (b) and IL-8 from donor 2 (c) and donor 3 (d) at 48 hours postseeding. Media and SIS ECM only samples were used as controls. Error bars represent SD of three biologic replicates with internal duplicates. *p < 0.05. (TIFF 4250 kb) [file 13287_2015_165_MOESM1_ESM.tiff]

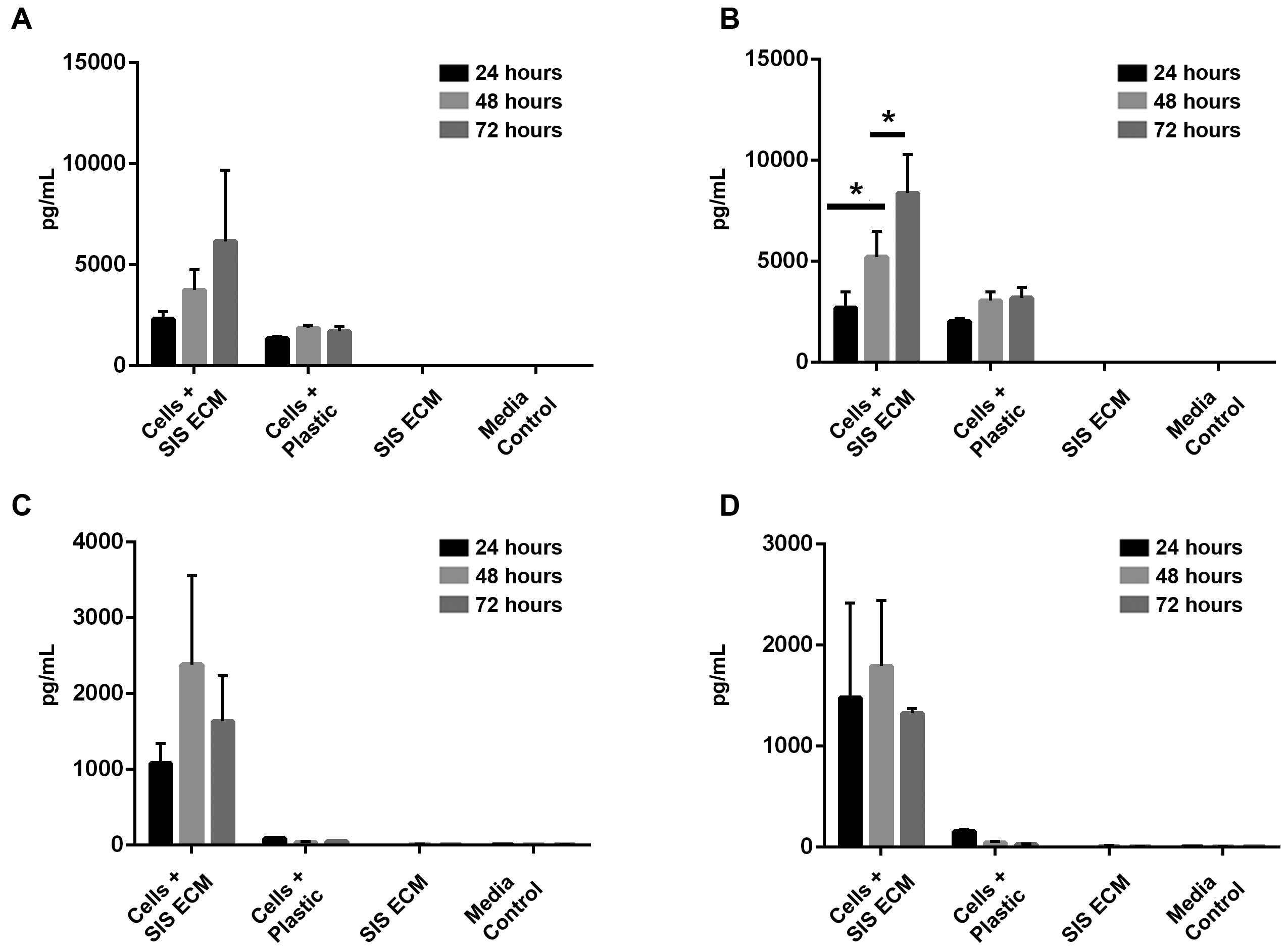

Supplement: Additional file 2: Figure S2. — ELISA measurement of VEGF and IL-8 release from donors 2 and 3 MSCs at three different time points. Human MSCs from donors 2 and 3 were seeded on SIS ECM or cell culture plastics and culture supernatant was used to quantify VEGF secretion from donor 2 (a) and donor 3 (b) and IL-8 from donor 2 (c) and donor 3 (d) at 24, 48 and 72 hours postseeding. Error bars represent SD of three biological replicates with internal duplicates. *p < 0.05. (TIFF 4707 kb) [file 13287_2015_165_MOESM2_ESM.tiff]

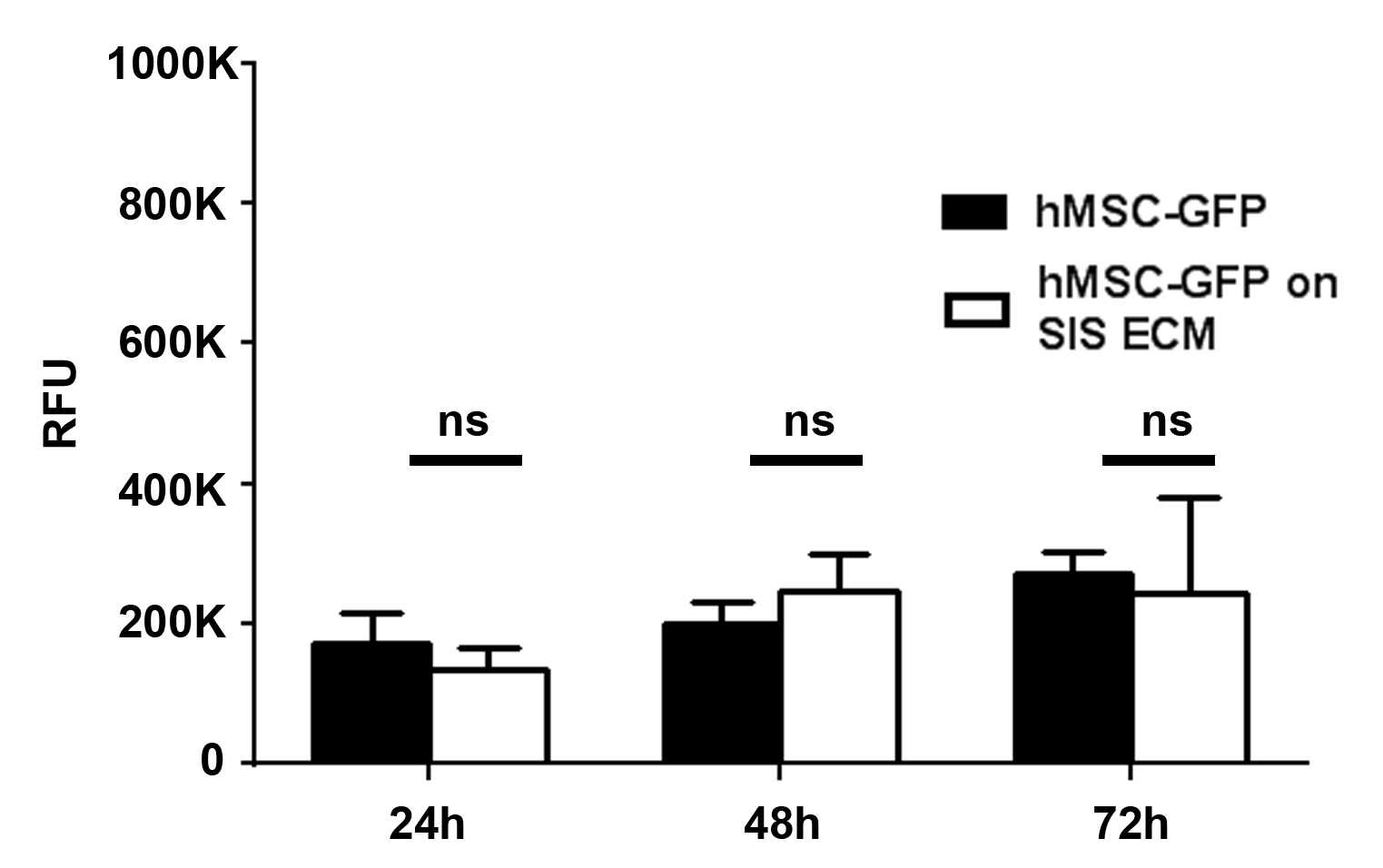

Supplement: Additional file 3: Figure S3. — Seeding on SIS ECM or culture plastic does not affect cell growth rates. MSCs from donor 2 overexpressing eGFP were seeded onto SIS ECM or tissue culture plastic and relative fluorescence intensity was measure after 24, 48 and 72 hours postseeding. Media and SIS ECM only controls were used to correct for background fluorescence. Error bars represent SD of six replicates. ns non-significant, RFU relative fluorescent units. (TIFF 1443 kb) [file 13287_2015_165_MOESM3_ESM.tiff]
